# Assessment of Genetic Parameters and Gene Action Associated with Heterosis for Enhancing Yield Characters in Novel Hybrid Rice Parental Lines

**DOI:** 10.3390/plants11030266

**Published:** 2022-01-19

**Authors:** Mahmoud M. Gaballah, Kotb A. Attia, Adel M. Ghoneim, Naeem Khan, Aziz F. EL-Ezz, Baochang Yang, Langtao Xiao, Eid I. Ibrahim, Abdullah A. Al-Doss

**Affiliations:** 1 Rice Research and Training Center, Field Crops Research Institute, Agricultural Research Center, Kafr El-Sheikh 33717, Egypt; mahmoudgab@yahoo.com (M.M.G.); adelrrtc.ghoneim@gmail.com (A.M.G.); abuelezz76@hotmail.com (A.F.E.-E.); 2 Department of Biochemistry, College of Science, King Saud University, P.O. Box 2455, Riyadh 11451, Saudi Arabia; 3 Department of Agronomy, Institute of Food and Agricultural Sciences, University of Florida, Gainesville, FL 32611, USA; naeemkhan@ufl.edu; 4 Hunan Provincial Key Laboratory of Phytohormones and Growth Development, Hunan Agricultural University, Changsha 410128, China; yangbaochang@foxmail.com (B.Y.); langtaoxiao@163.com (L.X.); 5 Biotechnology Lab., Plant Production Department, College of Food and Agriculture Sciences, King Saud University, Riyadh 11451, Saudi Arabia; eed4a@yahoo.com (E.I.I.); aaldoss@ksu.edu.sa (A.A.A.-D.)

**Keywords:** hybrid vigor, combining ability, gene action, principal components, hybrid rice

## Abstract

The technology of hybrid rice utilizing heterosis is an essential requirement for achieving food security. The current study was aimed at assessing the genetic parameters and the gene actions of 15 yield-component traits associated with heterosis, in 9 new parental lines of hybrid rice and their generated hybrids. Five cytoplasmic male sterile (CMS) lines were crossed with four restorer (R) lines using twenty generated line × tester designation hybrid combinations. The results revealed that all the traits were controlled by additive and non-additive gene actions. However, the additive variance was the main component of the total genotypic variance. Assessment of the general combining ability (GCA) detected the best combiners among the genotypes. The hybrid combinations that expressed the highest-positive specific combining ability (SCA) for grain-yield were detected. The correlation between the GCA and SCA was evaluated. The hybrid crosses with high-positive heterosis, due to having a better parent for grain yield, were detected. The principal component analysis (PCA) recorded the first four principal axis displayed Eigenvalues >1 and existing variation cumulative of 83.92% in the genotypes for yield component characteristics. Three-dimensional plots corresponding to the studied traits illustrated that the genotypes Guang8A × Giza181, Quan-9311A × Giza179, II-32A × Giza181, and II-32A × Giza179 are classified as possessing superior grain yield.

## 1. Introduction

Rice is the main cereal crop worldwide and feeds the majority of the world’s population. It is replacing wheat as the main source of achieving global food security. Heterosis in the first generation (F1) derived from hybrid rice parental lines provided 15–30% higher advanced yield than inbred varieties. In addition, heterosis means superior hybrid vigor in agronomic traits, grain yield, and other traits compared to their parents, as well as being more tolerant to stresses than inbred parents. Hybrid rice technology has provided a significant contribution to food security and employment opportunities. Hybrid rice has superior grain yield to inbred rice cultivars and is one of the most significant applications of heterosis in crops [1]. Rice is a self-pollinator, and most conventional rice cultivars are inbred lines. Hybrid seed production has been utilized using three-line or two-line systems [2]. The three-line method has a CMS line, a maintainer line (B), and a restorer line (R) [3]. Three-line hybrid rice played a significant role in developing rice production. Male sterility presence in CMS lines is governed by a single nuclear recessive gene or a pair of nuclear recessive genes sensitive to cytoplasm, including photoperiod or temperature, or their combination [4]. Filling up the gap among rice supply and demand as well as increasing productivity are the main plans to sustain rice production with decreased land area and fresh water resources [5]. Fortunately, by creating a functional cytoplasmic-genetic male sterility system for effective and economic seed production, the rice breeder overcomes these constraints. The effectiveness of hybrid rice technology depends on the genetic purity, time availability, and cost of hybrid seeds for farmers [6]. The progress of hybrid rice technology will decrease the cost of seeds and resilient tools, to decline the yield obstacles for rice. Combining ability analysis helps to identify superior parents to be used in breeding programs or to identify promising cross combinations for cultivar development. General combining ability (GCA) is directly related to the breeding value of a parent and is associated with additive gene effects, while specific combining ability (SCA) is the relative performance of a cross that is associated with non-additive gene action, predominantly contributed by dominance, epistasis, or genotype-environment interaction effects. Therefore, both GCA and SCA effects are important in the selection or development of breeding populations. In the effective hybrid breeding program, the line × tester is a powerful tool to differentiate between good and bad combiners to select suitable parental content. It provides reliable data on the nature and extended gene action as well as incorporates the possible effects of the genetic material [7]. The breeding value could be derived from the GCA and SCA for desired characteristics, from the genotypic and phenotypic values of the parent for highly inheritable traits [8]. The investigations on heterosis, the genetic basis of traits in different crops, shows that heterosis included partial, complete dominance, and over-dominance, as well as epistasis [9]. The over-dominance resulted in non-allelic interaction and linkage disequilibrium, and is a provider to heterosis [10]. Multivariate statistical analysis is widely used to summarize inherent differences among the genotypes. Principal component analysis (PCA) examines information comprised of numerous correlated, reliant quantities of traits as records [11]. The PCA summarized the data and resulted in new orthogonal parameters, named principal components. The PCA showed trends and decreases with the doubling of the data, as variance regularly completed in yield and its components’ trait [12]. The advantage of PCA is determining the significance of every variability dimension of a dataset [11]. The current investigation was aimed to estimate the genetic parameters, GCA, SCA, and the degree of gene actions of new CMS lines, new restorer testers, and their crosses. Moreover, to identify hybrid vigor levels in the generated hybrids.

## 2. Results

### 2.1. Analysis of Variance (ANOVA)

The variance analysis demonstrated significant differences among the studied genotypes for 15 yield-contributing traits. However, non-significant differences were observed among the replications for all characters except for PE, 1000-GW, AP-H, and PHA (Table 1). This reflected the inherent variability existence among all the genotypes. Analysis of variance due to parents versus hybrids (average heterosis) and line × tester (SCA) revealed a high-significant difference for all studied traits except for NNP (Table 1). This result reflected the interaction between the male and female lines that generated variable SCA effects, which perhaps is associated with the wide genetic variability among the parental lines.

### 2.2. Genetic Parameters and Gene Action Evaluation

Variances due to SCA were observed as much higher values than variances due to the GCA for all characteristics. This was interpreted to be the low magnitude of σ^2^GCA/σ^2^SCA ratios (Table 2). The ratio of GCA/SCA variance was less than one in all characters except for fertility percentage. The contribution to the expression of the 15 studied traits was varied among lines, testers, and their interaction (line × tester). The lines showed a high contribution to plant height and chlorophyll content. In contrast, the testers highly contributed to days to heading, flag leaf angle, and fertility %. Meanwhile, the contribution of their interaction (line × tester) was the highest based on the respective cross (Table 2). This indicates the additive gene action prevalence in those traits is referred to the lines contributed by more positive alleles, while the testers contributed by more positive alleles in days to heading and grain type. Genetic parameters evaluation for the studied characters observed that the dominance variance (*δ*^2^*D*) due to the SCA% for the traits was much higher than the additive variance (*δ*^2^*A*) due to the GCA% (Table 2). The heritability values in the broad sense (*h*^2^*b%*) were high for all the studied traits. This is contrary to the heritability values in the narrow sense (*h*^2^*n%*), which were much lower for all the traits except for phenotypic acceptability (Table 2). This finding indicates that the additive variance was the main component of the total genotypic variance.

### 2.3. Mean Performance

**Figure 1:**
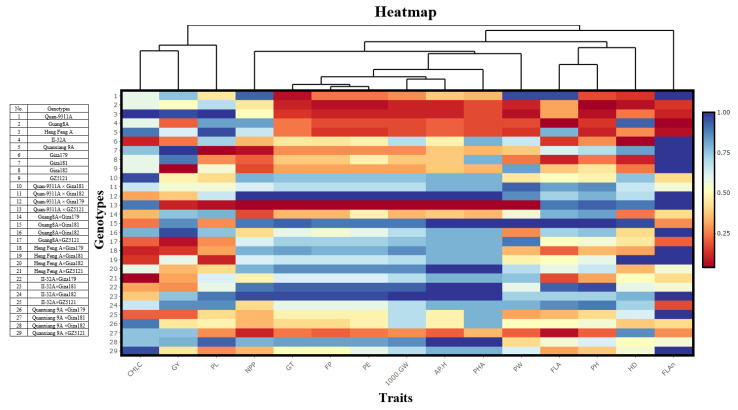
Heatmap analysis of 15 traits across 25 rice genotypes. Heatmap analysis data based on the main values of the traits of each genotype.

All genotypes recorded better mean performances in all studied characters. Moreover, the hybrids recorded higher performance in NPP, PW, 1000-GW, GY, APH, and PHA compared with the parental lines (Table S1). More specifically, 21 genotypes had the complete panicle exsertion (PE) percentage, while the partial and lowest values were recorded in the other genotypes. The highest panicle length (PL) (cm) was observed in crosses Quan-9311A × GZ5121 and II-32A × Giza179. The maximum number of panicles/plant (NPP) was obtained in crosses Guang8A × Giza18 and Quan-9311A × Giza 179. The heaviest panicle weight (PW) was found in crosses Quan-9311A × GizaI79 (Table S1). Maximum fertility percentage (FP) was found in crosses II-32A × Giza181 and Quan-9311A × Giza 179. The highest grain yield (GY) was found in crosses Quan-9311A × Giza 179 and II-32A × Giza179. The highest apparent heterosis (APH) was obtained in crosses Quan-9311A × Giza-179, II-32A × Giza179, and Quanxiang 9A × Giza182. Regardless of phenotypic acceptability (PHA), the desired mean values were found in crosses Quan-9311A × Giza179 and II-32A × Giza179. Generally, the hybrid combination Quan-9311A × Giza179 revealed the superiority in the yield contributed traits particularly for APH, PW, 1000-GW, FP, and GY (Table S1). Heatmap analysis data observed the significant differences among genotypes in vegetative traits as higher than that in reproductive traits (Figure 1). Moreover, the heatmap analysis classified the studied traits based on the genetic background of the genotypes. It showed a positive correlation in the pairs of traits such as (PH, FLAn), (HD, NNP), (FLA, PL), and (1000-GW, FP) (Figure 1).

### 2.4. Combining Ability Estimation

The effects of the GCA of 5 CMS and 4 restorer lines for 15 traits were analyzed (Table 3). The results showed that among the CMS lines, the line Quan-9311A exhibited as the best combiner due to the highly positive GCA value for DH, PL, and PE, whereas it was a good combiner for CHLC and NPP. The CMS line Guang8A revealed the highest GCA value for PH and FLA, while it was a good combiner in PL, GT, GY, and AP-H. The line Heng-FengA was recorded as the best combiner for PW, FP, PE, and AP-H. At the same time, it was a good combiner for NPP, 1000-GW, GY, and PHA traits (Table 3). In the case of line II-32A, which was revealed as the best combiner for PL, FLAn, 11000-GW, NPP, GY, APH, and PHA, whereas it was a good combiner for DH, PW, and FP. The CMS line Quanxiang 9A demonstrated as the best combiner for CHLC and GT (Table 3). Among the tester lines, the Giza179 identified as the best combiner only for PE, while it was a good combiner for PL, FLAn, FLA, FP, and 1000-GW (Table 3). The restorer Giza181 was the best combiner for DH, FLA, and GT, whereas it was a good combiner for CHLC, PW, FP, NPP, GY, AH, and PHA. On the other hand, Giza-182 was recorded as the best combiner for most characters such as PH, CHLC, PL, FLAn, FP, 1000-GW, NPP, GY, AH, and PHA; additionally, it was a good combiner for GT (Table 3). The SCA effect illustrated that 7 of 20 hybrid combinations performed highly negative and significant SCA effects of DH (Table 4). Moreover, 10 and 12 crosses showed significant and negative SCA effects for PH and FLAn, respectively. For chlorophyll content, 10 crosses showed significant positive values for SCA effect (Table 4). Eleven hybrid combinations showed significant and positive SCA effect for FLA, while ten crosses were significantly positive with SCA for PE. Likewise, 10 crosses exhibited significant-positive SCA effects for PL (Table 4). In terms of NPP, there were 9 hybrid crosses that reported a positive and significant SCA effect, whereas 10 crosses were significantly positive with an SCA effect for PW. Eleven hybrid crosses showed a significantly positive SCA effect for FP (Table 4). Similarly, 10 and 11 combinations showed a significantly positive SCA effect for 1000-GW and GY, respectively (Table 4). For GT and AH, 8 and 10 crosses showed positively significant SCA effect, respectively. The 13 out from 20 crosses reported significantly positive SCA effect to PHA (Table 4).

### 2.5. Correlation Analysis among Combining Abilities of Studied Characters

**Figure 2:**
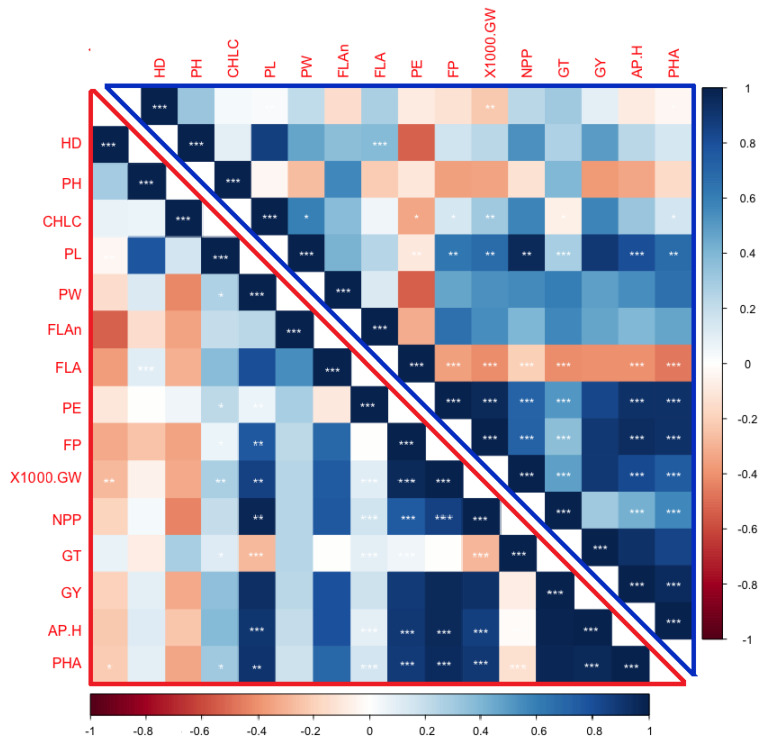
Corrplot depicting Pearson’s correlation between the general (upper triangle) and specific (lower triangle) combining ability for 15 yield traits across 29 genotypes. Red squares indicate a negative correlation; blue squares indicate a positive correlation; and white squares indicate no correlation. The asterisks indicate significant correlations using a two-tailed *t*-test (* and ** *p* < 0.05; and *** *p* < 0.01).

The distribution and correlation between GCA and SCA contributed significantly to different characteristics. The correlation can vary among GCAs or SCAs, from among the traits per se. The results showed that the correlation between the GCAs of two traits can be the same or vary from those between SCAs. Thus, our data recorded that the GCA PW was high-positive correlated with the GCA FP, 1000-GW, NPP, GT, APH, and PHA, while only correlated with GY, which was similar to the SCA PW of those traits (Figure 2). This demonstrated that there was a positive additive effect among these traits. Conversely, the GCA PW was high-negative with SCA GT, while it was high-positive with GCA GT, which indicated about the positive additive and negative non-additive gene actions between these two characters. Likewise, GCA GY showed positive correlation with all the traits except for negative correlation with CHLC and PE traits; similarly, SCA GY was positive correlated with those traits except for GT (Figure 2). In terms of the GCA, the APH trait was high-positive correlated with PW, FP, 1000-GW, NPP, and GT, while high-negative correlated with PE. Similarly, SCA APH was high-positive correlated with the SCA of those characters, except it showed no correlation with PE and GT (Figure 2). This finding showed that there are positive additive and negative non-additive effects among those traits. Thus, such correlation analysis indicated the importance of the GCA and SCA for understanding the genetic relationships among yield traits.

### 2.6. Heterosis Evaluation

Estimation values of better parent heterosis (BPH) were varied among the cross-combinations (Table 5). The BPH for HD showed high-significant negative in one cross II-32A × Giza181. In the aspect of PH, desirable high-significant negative heterosis was found for crosses Quanxiang9A × Giza181 and Quanxiang9A × GZ5121. Regarding the CHLC trait, four crosses showed high-significant positive heterosis. For the FLAn trait, 12 hybrids exhibited desirable negative-significant heterosis for this trait. On the other hand, eight hybrids had desirable positive-significant heterosis for FLA (Table 5). Eleven and seven out of twenty crosses confirmed high-significant positive BPH for PL and NPP, respectively. Sixteen hybrids showed significant positive BPH for FP, 1000-GW, and GT. Four crosses showed significant positive BPH for GY, including Guang8A × Giza179, Guang8A × Giza182, Heng Feng A × GZ5121, and II-32A × Giza179 (Table 5).

### 2.7. Multivariate Analysis

**Figure 3:**
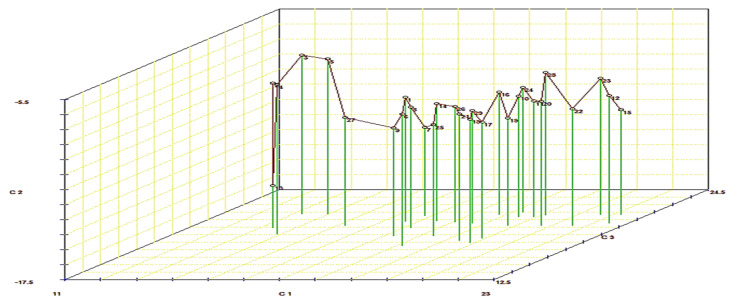
Three-dimensional scheme of yield potential (YP), yield stress (YS), and yield index (YI) for 29 rice genotypes; C1, C2, and C3 are the first, second, and third principal components, respectively.

**Figure 4:**
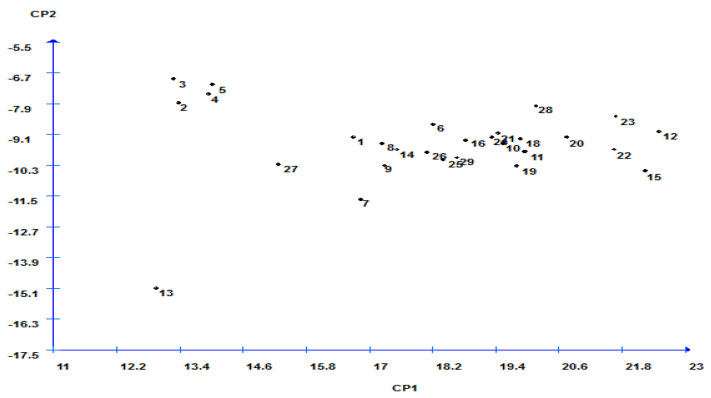
Biplot analysis for 15 traits across 29 genotypes; numbers 1, 2, 3… 29 represent the genotypes numbers, according to the serial number shown in Figure 1.

The selection relying on a grouping of characteristics may result in a more valuable criterion for improving rice to high yield potential. The first four components in the principal component analysis (PCA) with Eigen-values > 1 contributed 83.92% variability existing in the genotypes for yield component traits (Table 6). The remaining components with Eigen-values < 1 contributed 16.08% variability. The first PCA (PC1) was 50.35% of the difference and positive associated with all traits except PH, FP, and GY. Therefore, the first dimension was yield potential. Genotypes with high values of PC1 were high yielding. The PC2 represents 15.07% of the total variation and positive associated with most traits except FLAn, PH, FLA, 1000-GW, and NPP (Table 6). Consequently, PC1 featured PL (0.76) as the principal trait, followed by CHLC (0.35), FLAn (0.25), and PH (−0.41) in a negative direction. Meanwhile, PC2 was loaded mainly by characteristics, i.e., HD (0.69), PH (−0.58), CHLC (0.23), PW (0.23), PE (0.18), and 1000-GW (−0.11). The PC3 contributed negatively for HD (−0.42) and FLAn (−0.37) and positively for CHLC (0.71) and FLA (0.41). The PC4 was related with traits PH (0.74), CHLC (0.29), PW (0.47), and PE (0.26) (Table 6). To identify the relationship among studied traits and suitable high yield, a biplot and three-dimensional plots analysis were conducted (Figure 3 and Figure 4). These plots explained the ability of studied traits in induced groups. A three-dimensional diagram (Figure 3) could divide the genotypes into three groups: group-A included the crosses Guang8A × Giza181, Quan-9311A × Giza179, II-32A × Giza181, and II-32A × Giza179, which were high yield and expressed uniform superiority. Group-B included genotypes that showed medium performance for grain yield and uniformity. Group-C included genotypes with the lowest grain yield and declining uniformity (Figure 3).

## 3. Discussion

Utilization of heterosis through hybrid rice technology provides an effective solution to enhance rice yield. The current investigation was aimed to evaluate the genetic parameters, GCA, SCA, and gene actions of 5 CMS lines, 4 restorer testers, and their 20 crosses, to identify hybrid vigor levels in the generated hybrids.

### 3.1. Genetic Parameters and Gene Action Evaluation

The ANOVA results recorded significant differences among all tested traits across the genotypes (Table 1). This finding reflected the diversity of existence among these genotypes. Similarly, the variances due to parents versus hybrids and line × tester (SCA) showed significant differences for all characters except for NNP, which indicated wide genetic variability among parental lines. Our study indicated that the studied characters were controlled by additive and non-additive gene effects. These findings are consistent with the studies that have previously been reported [8,9,10,11,12,13,14,15,16,17,18,19]. An additive gene action effect played an important role in the early selection of the trait. Moreover, the additive and dominance (*σ*^2^_*A*_/*σ*^2^_*D*_) ratio could be used for genetic analysis in the heterotic population [1]. The genetic parameters evaluation for studied characters observed that the dominance variance (*δ*^2^_*D*_) due to the SCA% was much higher than the additive variance (*δ*^2^*A*) due to the GCA% for all studied traits (Table 2). This finding demonstrated the superiority of dominance gene action in the inheritance of characters [20]. Concurrently, the current results found that the dominance gene effect in chlorophyll content referred to the lines has positive alleles, while the testers contributed more positive alleles in days to heading and grain type, whereas the other studied traits were governed by line × tester interaction. These results were consistent with the data reported by [21,22]. The heritability (*h*^2^*b%*) values were much higher than those of the heritability (*h*^2^*n%*) for most of the studied traits (Table 2), which indicated that the additive variance was the main component of the total genotypic variance. Many previous studies revealed the effects of additive and non-additive gene actions, reflecting their importance for hybrid rice improvement [23,24].

### 3.2. Mean Performance and Combining Ability

Grain yield is a quantitative trait that is influenced by many other yield components, i.e., panicle number, panicle weight, grain filling, heading date, and 1000-grain weight. The current data showed that the hybrid combinations revealed higher performance in grain yield characters compared with the parental lines, which reflected the evidence of heterosis (Table S1). The hybrids Guang8A × Giza181, II-32A × Giza179, II-32A × Giza181, and Quan-9311A × Giza179 were superior to all parents in grain yield and its components. These combinations could be utilized as new hybrids for high yielding with good agronomic traits. It was reported that the large panicles and better flowering pattern, uniformity of panicle, flowering period concentration, and good panicle exsertion percentage may be highly effective for hybrid seed production [8]. Combining ability evaluation provides a guide-selection for crop breeders to select the proper parental lines for hybridization. In addition, it is a powerful tool to clarify the nature of gene action for desired characters [25]. It was reported that the GCA has a vital role in the heterotic degree of grain yield and its component characteristics [26]. Our current study observed that all parental lines were identified as the best combiner for at least one of the studied traits and a good combiner for a minimum two yield characteristics (Table 3). Among these, CMS line II-32A showed as the best combiner for PL, 1000-GW, NPP, GY, and APH, while, it was a good combiner for PW, and FP. On the other hand, the restorer Giza-182 was recorded as the best combiner for CHLC, PL, FP, 1000-GW, NPP, GY, and AH; additionally, it was a good combiner for GT (Table 3). These findings were better compared to previous reports that showed no single parental line was found to be the best or a good combiner for all yield-related characters [27,28,29]. Therefore, these studied parental lines can be used to improve grain yield and its component traits in hybrid-rice-breeding programs. The data of the current investigation revealed that the hybrid combinations revealed significantly positive SCA effects for a minimum of one yield characteristic. Among these, the crosses Quan-9311A × Giza179, Guang8A × Giza181, II-32A × Giza181, and Quanxiang 9A × GZ5121 showed significant high-positive SCA for FP, NPP, 1000-GW, and GY (Table 4). Thus these hybrid combinations could be utilized for improving heterosis and achieving a high-yielding genotype. The results demonstrated that no specific combination had positive SCA effects for all characters concurrently; this is consistent with those reported from previous results [24,25,26,27,28]. Our finding demonstrated that a certain cross-combination with a high positive SCA for different characters had both parents with a better GCA. This result revealed the role of the cumulative effects of additive × additive interactions of positive alleles [30,31]. In contrast, a hybrid combination with a high-positive SCA in a specific trait had at least one of its parents exhibiting poor GCA effects. This might be related to a good combiner parent expressing suitable additive effects and a poor combiner parent displaying epistatic effects [32,33]. At the same time, the parents with good general combiners did not always produce hybrid combinations with high-positive SCA. The cross Quanxiang 9A × GZ5121 exhibited high SCA for the GY and its contributed characters, although its parents recorded negative GCA for those traits (Table 3 and Table 4). Such a finding can happen because of the interaction between the positive and negative alleles of the parents. Similar findings were also found in previous studies [7,8,9,10,11,12,13,14,15,16,17,18,19,20,21,22,23,24,25,26,27,28].

### 3.3. Correlation and Heterosis Analysis

The influence of additive and non-additive gene actions of the characters is associated with the correlation between the GCAs or SCAs of those characters. Our current results showed that the correlation between the GCAs of two characters might be the same or different from those between the SCAs (Figure 2). This finding was consistent with previous published reports [34]. This indicated about the complex interaction of positive additive and negative non-additive gene action effects between those traits [31,32,33,34,35]. From these findings, we can understand the main roles of the GCA and SCA in explaining the genetic relationships among yield traits. Therefore, it is highly recommended for crop-breeders to consider the GCA, SCA, and the correlation between them for parental line selection aimed to produce heterotic hybrid genotypes. It is clear that the heterosis of grain yield depends on the quantitative effects of the dominance genes of those grain-yield related traits [24,25,26,27,28,29,30,31,32,33,34,35]. In our study, the crosses Guang8A × Giza179, Guang8A × Giza182, Heng Feng A × GZ5121, and II-32A × Giza179 had superior heterosis for grain yield (Table 5). Therefore, these crosses could be used for commercialization. It was reported that the crosses with high grain yield showed a high heterosis percent [9,10,11,12,13,14,15,16,17,18,19,20,21,22,23,24,25,26,27,28,29,30,31,32,33,34,35,36,37,38]. As it is known, negative heterosis for particular traits such as HD and PH is desirable in order to produce semi-dwarf and early maturing hybrids. Thus, in our results, the crosses that showed negative BPH in HD and PH traits could be utilized in a breeding program for producing early and semi-dwarf hybrids. Principal component analysis is a technique for reducing high-dimensional vector data to lower-dimensional data. It clarifies the divergence genetically among the genotypes for all traits. Each component of the data was considered with Eigenvalues, more than one controlling at least 10% of the variation. Superior Eigenvalues are the greatest qualities in the principal components. Our data observed that the four principal axis displayed Eigenvalues >1 and existing variation of 83.92%. This indicates that the identified characters within these components show major impacts on phenotypic genotypes. These results were consistent with previous data reported by [39,40]. Principal component analysis showed that PC1 and PC2 were governed by yield related traits, viz., PL, FLAn, CHLC, DH, PH, PW, PE, and 1000-GW. Therefore, the traits are the most significant to classify the present variation in the genotypes. Consequently, intensive selection procedures may be planned to bring about rapid development of the dependent variable, i.e., grain yield, by selecting lines from PC1 and PC2 [12,13,14,15,16,17,18,19,20,21,22,23,24,25,26,27,28,29,30,31,32,33,34,35,36,37,38,39,40,41,42].

## 4. Materials and Methods

### 4.1. Genetic Materials

A total of 20 test crosses were generated from 5 CMS lines and 4 restorer lines, testing by line × tester mating as described by [13]. The parents (Table 7) were grown thrice with an interval of 10 days to synchronization in flowering for crossing in Sanya, Hainan Province, China, during the 2017 season. The parents and their derived combinations were evaluated at the experimental farm of the Winall Thriving Seed Company (WTSC) during the 2018 season in Hefei, Anhui Province, China. In the 2017 rice-growing season, one-month old seedlings are transplanted into the permanent field in 5 rows with 10 plants/row at a distance of 20 cm × 20 cm. The F1 seeds were harvested at 25 days from pollination. The F1 seeds and their parents were sown on 1 May 2018. The seedlings were transplanted 30 days later into the permanent field with one seedling/hill. A randomized complete block design (RCBD) with three replications was used.

Each genotype was planted in 15 rows with 20 plants per row at a distance of 20 cm 15 cm. Each test entry had 15 rows of 2 m length; the genotype area was 6 m^2^. The seeds were soaked for two days and covered for one day after radically sprouting. The seeds were planted in the nursery and covered with mud. All procedures were done according to IRR-2002. All recommended practices for planting, transplanting, N, P, K, and Zn fertilizers, water management, and plant protection were done as recommended.

### 4.2. Field Evaluation

At harvest time, all the selected panicles from 10 randomly plants were harvested, dried, and threshed. Grain yield (t ha^−1^, GY) for every genotype was noted from 25 plants, adjusted into 14% moisture content, and converted into t ha-1. Studied agronomic trait and days to 50% heading (HD) were measured from the sowing date until 50% of the plot was flowering, plant height (cm; PH) was measured from soil surface until the upper main panicle for each plant; and SPAD-chlorophyll values (CHLC; CC) were determined by using a chlorophyll analytical apparatus, chlorophyll meter SPAD-502 Minolta Camera Co., Ltd., Japan. Five flag leaves were measured from the widest part of the leaf of the main culm for each entry in all replications. Flag leaf angle (°; FLAn) was measured at heading by separating the main tiller from the rest of the plant; the tiller is immediately placed against a vertical board covered with paper so, while the culm is the vertical axis with the leaves drooping normally from the axis, marking the positions of the tip and collar of each leaf on the paper and drawing a line between the two points and measure the angle between the line and vertical axis with a protractor; flag leaf area (cm^2^; FLA) is calculated in cm^2^ in the whole flowering period following the formulation (flag leaf area (cm^2^) = K × leaf length(cm) × maximum width(cm)), where K (0.75); for panicle length (cm; PL), the main panicle was measured from the panicle base up to a piculus of the upper most spikelet of the panicle (IRRI, 1996); the number of panicles plant^−1^ (NPP) was determined by counted the number of the number of panicles/plant at harvesting; for panicle weight (g; PW), each panicle was weighed and recorded; fertility percentage (%; FP) was accounted for by the number of fertility spikelets divided by the whole-formed spikelets on the panicle; 1000-grain weight (g; 1000-GW) was random selected from 1000-grains and weighed. All data were recorded according to a standard evaluation system (IRRI, 2002). Panicle exsertion (PE) refers to the percentage of the panicle exerted from the flag leaf sheath to total panicle length as follows: PE (%) = Length (cm) of exerted panicle/Total length (cm) of panicle × 100.

Grain type (GT) as per 1–9 scale, where the 1, 3, 5, 9 scale is based on length: width ratio, viz., slender is >3.0; medium is 2.1 to 3.0; bold is 1.1 to 2.0; and round is <1.0, respectively. Apparent heterosis (AP-H) refers to the superiority of a hybrid compared to its parents or a check cultivar visually recorded that then expresses as strong vegetative growth in the field. Phenotypic acceptability (PHA) of pollen sterility or fertility were observed and lines were assessed on an individual plant for a 1–9 scale of phenotypic acceptability, where 1 is unacceptable, 3 is poor, 5 is fair, 7 is good and 9 is excellent, according to [14].

### 4.3. Combining Ability Analysis

GCA and SCA effects and their variances were determined by line × tester analysis [15]. The analysis was done using the Agrobase statistical software package. Calculated dominance and additive genetic variances (*σ*^2^_*A*_ and *σ*^2^_*D*_) were with an inbreeding coefficient (*F* = 1), whereas each tester and line was inbred. The GCA and SCA effect significance tests were done by using a *t*-test. The relative weight of assessed additive vs. non-additive types of gene actions was defined by [16]. $\left(\sigma {\;}_{gca}^{2}/\sigma_{sca}^{2}\right),\;\mathrm{and}\;(\sigma_{D}^{2}/\sigma_{A}^{2}){\;}^{1/2}$ Ratios are additive vs. non-additive types of gene actions, rating relative weight.

### 4.4. Estimation of Heterosis

Better parent heterosis (BPH) or heterobeltiosis was calculated in spite of the increased or decreased percentage of the F1 hybrid than its better parent (BP) [17]. BPH (%) = [(F1)^−^ − (BP)^−^/BP] × 100. Better heterosis significance was determined by a *t*-test. BP ((t))^−^ = (F1)^−^ − BP/√((2/r)EMS), where F1 is the mean of the F1 hybrid for a character, BP is the better parent mean in the cross, and EMS is the mean square error.

### 4.5. Principal Component and Statistical Analysis

The PCA was done by subroutine EIGEN using Genes software [18]. The analysis of variance was layout by using a general linear model: Yijk = μ + Pij + rk + eijk. Where Yijk = value observed, µ = mean of the population, Pij = effect means of the ijth genotype, rk = kth affect replication, and eijk is experimental error assumed with ijkth observation and presumed to have a normal and independent distribution with the variance and mean. The comparisons mean between genotypes was evaluated by Tukey’s test (*p* < 0.05). The correlation coefficient was done between values of various characteristics by Pearson’s method. The correlation between the values of the characters was performed by Pearson correlation coefficients and plotted via the packages corrplot and Performance Analytics [8].

## 5. Conclusions

The current study estimated the genetic parameters, GCA, SCA, and nature of gene actions related to the heterosis of 15 yield-component characters in 9 parental lines with their hybrid crosses. From the data analysis, we found that all studied characteristics were governed by both additive and non-additive gene actions. The best combiners among the parental lines were identified; in addition, the hybrid combinations that recorded the highest GY were detected. Moreover, the correlation between the GCA and SCA was clarified. The hybrid crosses with high-positive heterosis due to a better parent for grain yield were named. Three-dimensional plots corresponding to studied traits illustrated that the genotypes Guang8A × Giza181, Quan-9311A × Giza179, II-32A × Giza181, and II-32A × Giza179 recorded high grain yields.

## Figures and Tables

**Table 1:** Analysis of variance for 15 yield and contributing traits across 29 genotypes.

Source of Variance	Reps	Genotypes	Parents	Crosses	Par. vs. Crosses	Lines	Testers	Lines × Testers	Residual	Total
Df	2	28	8	19	1	4	3	12	56	86
Days to heading (day)	0.05	132.8 **	257.06 **	86.804 **	13.995 **	45.173 **	273.42 **	54.02 **	0.46	
Plant height (cm)	0.05	348.07 **	595.05 **	214.55 **	909.05 **	446.51 **	88.26 **	168.81 **	1.85	
Chlorophyllcontent (SPAD)	0.40	15.27 **	11.699 **	17.244 **	6.40 **	47.28 **	7.03 **	9.784 **	0.18	
Panicle length (cm)	0.04	14.73 **	23.00 **	10.40 **	30.96 **	18.52 **	6.36 **	8.70 **	0.07	
Panicle weight (g)	0.01	5.211 **	0.329 **	4.421 **	59.29 **	3.17 **	5.65 **	4.52 **	0.02	
Flag leaf angle (°)	0.04	332.46 **	484.75 **	269.67 **	307.24 **	161.49 **	640.07 **	213.13 **	0.12	
Flag leaf area (cm^2^)	0.07	967.10 **	979.57 **	868.67 **	2737.4 **	128.76 **	532.55 **	1199.3 **	1.55	
Panicle exsertion (%)	0.31 **	63.67 **	150.74 **	2.411 **	531.11 **	1.90 **	0.80 **	2.98 **	0.01	
Fertility percentage (%)	0.07	717.95 **	173.73 **	984.37 **	9.725 **	662.30 **	1222.8 **	1032.1 **	0.2	
1000-grain weight (g)	0.01 **	2.44 **	1.41 **	1.81 **	22.69 **	1.21 **	1.72 **	2.03 **	0.2	
Number of panicles plant^−1^	0.02	19.61 **	3.39 **	17.53 **	1.00 ^ns^	6.51 **	24.77 **	12.00 ^ns^	0.11	
Grain type	0.03	6.95 **	10.12 **	5.94 **	0.70 **	3.21 **	20.87 **	3.12 **	0.01	
Grain Yield t/ha	0.04	36.87 **	11.39 **	28.94 **	391.60 **	22.17 **	25.41 **	32.08 **	0.01	
Apparent heterosis	0.008 **	8.956 **	3.07 **	6.946 **	94.23 **	3.88 **	8.17 **	7.661 **	0.03	
Phenotypic acceptability	0.01 **	3.47 **	2.069 **	2.66 **	29.98 **	1.64 **	3.73 **	2.73 **	0.02	

^ns^ and ** are not significant and are highly significant at 0.01 probability, respectively.

**Table 2:** Genetic parameters for 15 yield characteristics of tested genotypes.

Traits	Cov H.S (Lines)	Cov H.S (Tester)	Cov F.S (Line × Tester)	δ^2^ GCA	δ^2^ SCA	δ^2^GCA/δ^2^SCA	*δ* ^2^ *D*	*δ* ^2^ *A*	(*σ*^2^*D*/*σ*^2^*A*) 1/2	EV	GV	PV	H^2^b	H^2^n
Days to heading (day)	−0.74	14.63	40.29	0.96	17.85	0.05	17.9	1.91	3.06	0.46	44.11	44.57	98.97	4.29
Plant height (cm)	23.14	−5.37	76.23	1.34	55.65	0.02	55.7	2.67	4.57	1.85	115.41	117.26	98.42	2.28
Chlorophyll content (SPAD)	3.13	−0.18	6.84	0.22	3.20	0.07	3.2	0.44	2.70	0.18	5.03	5.21	96.55	8.45
Panicle length (cm)	0.82	−0.16	3.66	0.05	2.88	0.02	2.9	0.10	5.37	0.07	4.89	4.96	98.59	2.02
Panicle weight (g)	−0.11	0.08	1.48	0.00	1.50	0.00	1.5	0.01	12.25	0.02	1.73	1.75	98.86	0.57
Flag leaf angle (°)	−4.30	28.46	111.06	1.65	71.00	0.02	71.0	3.30	4.64	0.12	110.78	110.90	99.89	2.98
Flag leaf area (cm^2^)	−89.22	−44.45	215.88	−9.65	399.26	−0.02	399.3	1.01	19.88	1.55	321.85	323.40	99.52	0.31
Panicle exertion (%)	−0.09	−0.15	0.65	−0.02	343.97	0.00	1.0	0.03	5.74	0.01	21.22	21.23	99.95	0.14
Fertility percentage (%)	−30.82	12.71	325.46	−1.39	1.04	−1.34	344.0	0.99	18.64	0.20	239.25	239.45	99.92	0.41
1000-grain weight (g)	−0.1	0.001	0.6	0.01	106.9	0.01	1.0	0.1	26.5	0.2	0.7	0.9	78.87	10.56
Number of panicles plant^-1^	−1.07	0.36	5.65	−0.05	0.06	−0.89	6.4	0.11	7.65	0.11	6.50	6.61	98.34	1.66
Grain type	0.01	1.18	2.94	0.08	2.54	0.03	1.0	0.17	2.47	0.01	2.31	2.32	99.57	7.32
Grain yield t/ha	−0.83	−0.44	8.94	−0.09	0.90	−0.10	10.7	1.01	3.25	0.01	12.29	12.30	99.92	8.21
Apparent heterosis	−0.32	0.03	2.21	−0.02	3.96	−0.01	2.6	1.01	1.59	0.03	2.98	3.01	99.00	33.61
Phenotypic acceptability	−0.09	0.07	0.90	0.00	0.99	0.00	0.9	1.11	0.91	0.02	1.15	1.17	98.29	94.87

**Table 3:** General combining ability effects of lines and testers for studied traits.

**Lines**	**Days to Heading (Day)**	**Plant Height (cm)**	**Chlorophyll Content (SPAD)**	**Panicle Length (cm)**	**Panicle Weight (g)**	**Flag Leaf Angle (°)**	**Flag Leaf Area (cm^2^)**	**Panicle Exsertion (%)**
Quan-9311A	2.57 **	3.30 **	1.54 **	0.84 **	0.17 **	−0.85 **	−4.26 **	0.39 **
Guang8A	−1.28 **	6.29 **	−0.43 **	0.83 **	−0.18 **	0.33 **	4.16 **	−0.44 **
Heng Feng A	−1.32 **	−5.43 **	−2.95 **	−0.59 **	0.43 **	−5.65 **	−0.54	0.39 **
II-32A	1.60 **	3.41 **	−0.27 *	0.84 **	0.40 **	4.10 **	2.19 **	−0.38 **
Quanxiang 9A	−1.56 **	−7.57 **	2.11 **	−1.93 **	−0.81 **	2.07 **	−1.55 **	0.05*
LSD0.05	0.32	0.65	0.20	0.12	0.07	0.16	0.59	0.05
LSD0.01	0.46	0.91	0.29	0.17	0.10	0.23	0.83	0.06
Testers								
Giza179	−3.92 **	−0.27	0.09	0.22 **	−0.09 **	0.37 **	2.92 **	0.29 **
Giza181	6.02 **	1.74 **	−0.30 **	−0.60 **	0.19 **	−1.96 **	6.75 **	−0.05 **
Giza182	−1.68 **	1.87 **	0.91 **	0.82 **	0.68 **	8.63 **	−3.16 **	−0.27 **
GZ5121	−0.41 **	−3.33 **	−0.70 **	−0.43 **	−0.79 **	−7.04 **	−6.51 **	0.03
LSD 5%	0.28	0.58	0.18	0.11	0.06	0.14	0.53	0.04
LSD 1%	0.40	0.82	0.26	0.16	0.08	0.20	0.75	0.06
**Lines**	**Fertility Percentage (%)**	**1000-Grain Weight (g)**	**Number of Panicles Plant^−1^**	**Grain Type**	**Grain Yield t ha^−1^**	**Apparent Heterosis**	**Phenotypic Acceptability**	
Quan-9311A	−11.56 **	−1.4 **	0.19 **	−0.30 **	−0.84 **	−0.70 **	−0.55 **	
Guang8A	2.67 **	0.3 **	−0.09	0.45 **	0.17 **	0.05 **	−0.05 **	
Heng Feng A	6.56 **	0.7 **	0.41 **	−0.58 **	0.76 **	0.55 **	0.20 **	
II-32A	5.30 **	1.1 **	0.70 **	−0.20 **	1.70 **	0.55 **	0.45 **	
Quanxiang 9A	−2.98 **	−0.6 **	−1.21 **	0.63 **	−1.79 **	−0.45 **	−0.05 **	
LSD0.05	0.21	0.01	0.16	0.03	0.04	0.001	0.001	
LSD0.01	0.30	0.02	0.22	0.05	0.05	0.003	0.004	
**Testers**								
Giza179	2.94 **	0.5 **	−0.04	−0.40 **	0.01	−0.10 **	−0.05 **	
Giza181	3.76 **	0.01	0.56 **	1.13 **	0.37 **	0.10 **	0.15 **	
Giza182	6.63 **	1.01 **	1.24 **	0.75 **	1.37 **	0.90 **	0.55 **	
GZ5121	−13.33 **	−1.5 **	−1.76 **	−1.48 **	−1.75 **	−0.90 **	−0.65 **	
LSD 5%	0.19	0.01	0.14	0.03	0.03	0.001	0.001	
LSD 1%	0.27	0.02	0.20	0.04	0.05	0.002	0.003	

ns and ** are not significant and are highly significant at 0.01 probability, respectively.

**Table 4:** Specific combining ability effects of hybrid crosses for studied traits.

**Combinations**	**Days to Heading (Day)**	**Plant Height (cm)**	**Chlorophyll Content (SPAD)**	**Panicle Length (cm)**	**Panicle Weight (g)**	**Flag Leaf Angle (°)**	**Flag Leaf Area (cm^2^)**	**Panicle Exsertion (%)**
Quan-9311A × Giza181	3.49 **	−6.99 **	1.66 **	−1.10 **	0.38 **	−1.86 **	3.51 **	−0.44 **
Quan-9311A × Giza182	−8.43 **	0.50	0.02	1.22 **	−0.16 *	5.42 **	12.77 **	−0.17 **
Quan-9311A × Giza179	0.62 **	−2.25 **	−3.22 **	−0.71 **	1.55 **	0.58 **	11.78 **	0.45 **
Quan-9311A × GZ5121	4.33 **	8.74 **	1.54 **	0.59 **	−1.78 **	−4.15 **	−28.06 **	0.15 **
Guang8A × Giza179	−3.23 **	−1.98 **	−0.27	−0.09	−1.23 **	11.81 **	−3.61 **	0.39 **
Guang8A × Giza181	5.57 **	13.51 **	−0.86 **	1.23 **	0.98 **	−5.67 **	6.45 **	−0.29 **
Guang8A × Giza182	0.24	−6.12 **	1.79 **	−0.70 **	−1.05 **	−5.55 **	−21.61 **	1.28 **
Guang8A × GZ5121	−2.58 **	−5.42 **	−0.66 **	−0.44 **	1.30 **	−0.58 **	18.77 **	−1.39 **
Heng Feng A × Giza179	−0.99 **	−2.31 **	−0.96 **	−0.92 **	0.27 **	2.94 **	14.79 **	−0.11*
Heng Feng A × Giza181	5.77 **	1.23	0.45 *	1.15 **	−0.47 **	−3.42 **	−9.22 **	0.23 **
Heng Feng A × Giza182	−2.74 **	2.78 **	2.44 **	0.28 *	−0.16 *	−5.32 **	−9.79 **	0.05
Heng Feng A × GZ5121	−2.04 **	−1.70 *	−1.92 **	−0.52 **	0.36 **	5.80 **	4.22 **	−0.18 **
II-32A × Giza179	0.51 **	5.90 **	−0.60 **	0.44 **	0.95 **	−6.81 **	−1.24 *	0.25 **
II-32A × Giza181	−3.53 **	−3.11 **	0.81 **	0.72 **	0.91 **	11.07 **	17.65 **	1.00 **
II-32A × Giza182	2.41 **	2.26 **	0.62 **	−0.21	−1.18 **	−4.87 **	0.74	−2.16 **
II-32A × GZ5121	0.61	−5.05 **	−0.83 **	−0.95 **	−0.69 **	0.60 **	−17.16 **	0.92 **
Quanxiang 9A × Giza179	0.22	5.38 **	0.18	1.67 **	−0.37 **	−6.08 **	−13.45 **	−0.10*
Quanxiang 9A × Giza181	0.63 **	−12.13 **	−0.42 *	−4.31 **	−1.26 **	−7.40 **	−27.66 **	−0.78 **
Quanxiang 9A × Giza182	−0.53	3.32 **	−1.63 **	1.33 **	0.83 **	15.16 **	18.88 **	0.38 **
Quanxiang 9A × GZ5121	−0.32	3.43 **	1.87 **	1.32 **	0.80 **	−1.67 **	22.23 **	0.49 **
LSD 5%	0.64	1.29	0.41	0.25	0.13	0.32	1.18	0.09
LSD 1%	0.91	1.83	0.57	0.35	0.19	0.46	1.67	0.13
**Combinations**	**Fertility Percentage (%)**	**1000-Grain Weight (g)**	**Number of Panicles Plant^−1^**	**Grain Type**	**Grain Yield t ha^−1^**	**Apparent Heterosis**	**Phenotypic Acceptability**	
Quan-9311A × Giza181	16.53 **	1.7 **	0.38 *	1.22 **	1.65 **	1.10 **	0.55 **	
Quan-9311A × Giza182	13.70 **	1.5 **	−0.60 **	−0.02	0.88 **	0.90 **	0.35 **	
Quan-9311A × Giza179	16.86 **	2.6 **	4.11 **	−0.75 **	3.57 **	1.10 **	0.95 **	
Quan-9311A × GZ5121	−47.09 **	−5.9 **	−3.89 **	−0.45 **	−6.11 **	−3.09 **	−1.84 **	
Guang8A × Giza179	−12.77 **	−2.2 **	−1.84 **	1.16 **	−3.15 **	−1.65 **	−0.95 **	
Guang8A × Giza181	3.49 **	1.3 **	1.56 **	−0.17 **	2.55 **	1.15 **	0.85 **	
Guang8A × Giza182	−6.41 **	−1.1 **	−2.11 **	0.52 **	−1.48 **	−0.65 **	−0.55 **	
Guang8A × GZ5121	15.68 **	2.01 **	2.39 **	−1.50 **	2.09 **	1.15 **	0.65 **	
Heng Feng A × Giza179	−1.59 **	0.01	0.66 **	−1.14 **	0.30 **	−0.15 **	−0.20 **	
Heng Feng A × Giza181	−5.30 **	−0.5 **	−0.94 **	0.46 **	−0.87 **	−0.35 **	−0.40 **	
Heng Feng A × Giza182	−4.78 **	−0.5 **	−0.11	−0.57 **	−0.55 **	−0.15 **	0.20 **	
Heng Feng A × GZ5121	11.67 **	1.01 **	0.39*	1.25 **	1.12 **	0.65 **	0.40 **	
II-32A × Giza179	0.77 **	0.2 **	1.52 **	−1.01 **	1.38 **	0.85 **	0.55 **	
II-32A × Giza181	1.87 **	1.1 **	2.28 **	0.28 **	2.03 **	0.65 **	0.35 **	
II-32A × Giza182	−9.04 **	−1.9 **	−2.90 **	0.87 **	−3.01 **	−1.15 **	−1.05 **	
II-32A × GZ5121	6.40 **	0.6 **	−0.90 **	−0.14 **	−0.39 **	−0.35 **	0.15 **	
Quanxiang 9A × Giza179	−2.94 **	0.3 **	−0.72 **	−0.23 **	−0.18 **	−0.15 **	0.05 **	
Quanxiang 9A × Giza181	−13.76 **	−3.3 **	−2.31 **	−0.55 **	−4.59 **	−2.34 **	−1.15 **	
Quanxiang 9A × Giza182	3.37 **	0.8 **	1.01 **	−0.07 *	1.48 **	0.85 **	0.45 **	
Quanxiang 9A × GZ5121	13.33 **	2.3 **	2.01 **	0.85 **	3.29 **	1.65 **	0.65 **	
LSD 5%	0.43	0.01	0.31	0.07	0.07	0.006	0.001	
LSD 1%	0.60	0.02	0.44	0.10	0.10	0.01	0.002	

* and ** at *p* = 0.05 and 0.01, respectively, based on *t*-test.

**Table 5:** Heterosis over better parent for studied traits.

**Genotypes**	**Days to Heading (Day)**	**Plant Height (cm)**	**Chlorophyll Content (SPAD)**	**Panicle Length (cm)**	**Panicle Weight (g)**	**Flag Leaf Angle (°)**	**Flag Leaf Area (cm^2^)**	**Panicle Exsertion (%)**
Quan-9311A × Giza181	30.26 **	11.86 **	7.28 **	0.00	−12.16 **	−0.33 **	−6.09 **	25.00 **
Quan-9311A × Giza182	0.77	3.96 **	−0.32	385.29 **	3.93 **	−0.41 **	−0.87 **	34.67 **
Quan-9311A × Giza179	16.22 **	20.19 **	−2.93 **	105.39 **	−9.46 **	0.00	−2.61 **	75.00 **
Quan-9311A × GZ5121	19.74 **	12.19 **	5.00 **	−49.00 **	−62.53 **	0.00	−2.45 **	−31.58 **
Guang8A × Giza179	14.28 **	20.10 **	−2.50 **	49.19 **	1.46	−0.33 **	7.67 **	0.00
Guang8A × Giza181	4.22 **	27.62 **	−7.32 *	191.18 **	26.57 **	−1.35 **	2.63 **	55.56 **
Guang8A × Giza182	10.46 **	19.27 **	4.73 **	72.05 **	−15.41 **	0.00	7.67 **	10.00 **
Guang8A × GZ5121	5.50 **	4.76 **	−5.49 **	−36.06 **	11.22 **	−2.37 **	0.93 **	31.58 **
Heng Feng A × Giza179	17.59 **	7.67 **	−16.83 **	−33.33 **	20.56 **	0.00	−0.98 **	30.00 **
Heng Feng A × Giza181	4.40 **	3.84 **	−14.46 **	117.80 **	−3.17 **	0.00	−2.77 **	33.33 **
Heng Feng A × Giza182	6.35 **	16.34 **	−6.98 **	33.33 **	−3.04 **	−0.41 **	5.96 **	35.00 **
Heng Feng A × GZ5121	6.16 **	5.15 **	−4.93 **	−38.38 **	−13.75 **	−0.33 **	−4.67 **	15.79 **
II-32A × Giza179	24.30 **	54.19 **	−2.93 **	−33.33 **	2.02	−0.41 **	13.05 **	41.40 **
II-32A × Giza181	−1.55 **	45.30 **	−2.81 **	593.00 **	40.04 **	0.00	0.83 **	72.22 **
II-32A × Giza182	17.34 **	52.28 **	2.18 **	102.02 **	19.97 **	−3.38 **	16.33 **	10.00 **
II-32A × GZ5121	13.55 **	36.42 **	−5.49 **	−20.00 **	−37.94 **	0.00	−0.93 **	5.26 **
Quanxiang 9A × Giza179	19.08 **	13.40 **	0.00	−37.71 **	−20.21 **	−0.33 **	−1.82 **	0.00
Quanxiang 9A × Giza181	2.98 **	−16.74 **	−2.38 **	191.18 **	−31.57 **	−1.35 **	−27.26 **	0.00
Quanxiang 9A × Giza182	9.03 **	14.67 **	−2.38 **	223.23 **	44.97 **	−0.41 **	−0.85 **	30.00 **
Quanxiang 9A × GZ5121	8.13 **	−2.78 *	2.18 **	−37.37 **	8.30 **	0.00	−5.45 **	15.79 **
LSD 5%	1.11	2.23	0.70	0.56	2.03	0.16	0.42	0.54
LSD 1%	1.47	2.96	0.93	0.74	2.71	0.21	0.56	0.71
**Genotypes**	**Fertility Percentage (%)**	**1000-Grain Weight (g)**	**Number of Panicles/plant**	**Grain Type**	**Grain Yield t ha^−1^**	**Apparent Heterosis**		**Phenotypic Acceptability**
Quan-9311A × Giza181	85.64 **	2.31 **	−1.21 **	33.33 **	−8.57 **	14.29 **		0.00
Quan-9311A × Giza182	116.45 **	18.18 **	−3.40 **	74.31 **	−13.33 **	33.33 **		14.29 **
Quan-9311A × Giza179	192.31 **	12.00 **	3.18 **	90.43 **	−21.59 **	50.00 **		12.50 **
Quan-9311A × GZ5121	−45.45 **	−29.17 **	−88.52 **	−94.42 **	−42.50 **	−50.00 **		−28.57 **
Guang8A × Giza179	−10.89 **	−6.15 **	−12.91 **	−17.97 **	6.67 **	−14.29 **		−12.50 **
Guang8A × Giza181	165.31 **	24.55 **	6.83 **	123.72 **	−6.58 **	50.00 **		28.57 **
Guang8A × Giza182	41.03 **	4.00 **	−1.30 **	31.83 **	2.54 **	33.33 **		0.00
Guang8A × GZ5121	120.30 **	10.83 **	1.16 **	61.49 **	−42.67 **	33.33 **		14.29 **
Heng Feng A × Giza179	93.07 **	3.85 **	3.31 **	36.72 **	−44.71 **	14.29 **		0.00
Heng Feng A × Giza181	113.36 **	18.18 **	0.00	71.50 **	−11.13 **	33.33 **		14.29 **
Heng Feng A × Giza182	117.95 **	8.00 **	3.88 **	53.81 **	−24.71 **	50.00 **		12.50 **
Heng Feng A × GZ5121	100.00 **	8.33 **	8.75 **	54.95 **	18.31 **	33.33 **		14.29 **
II-32A × Giza179	125.25 **	6.15 **	0.55	64.06 **	2.54 **	28.57 **		12.50 **
II-32A × Giza181	196.85 **	27.27 **	2.65 **	142.36 **	−8.86 **	50.00 **		28.57 **
II-32A × Giza182	64.10 **	4.00 **	−6.18 **	31.83 **	−1.08 **	33.33 **		0.00
II-32A × GZ5121	34.67 **	8.33 **	−11.15 **	45.26 **	0.00	16.67 **		14.29 **
Quanxiang 9A × Giza179	0.00	0.00	−10.67 **	−4.30 **	−13.92 **	0.00		0.00
Quanxiang 9A × Giza181	−12.49 **	0.00	−21.91 **	−44.07 **	−8.86 **	−16.67 **		0.00
Quanxiang 9A × Giza182	105.13 **	8.00 **	0.56	46.48 **	−2.29 **	50.00 **		12.50 **
Quanxiang 9A × GZ5121	51.52 **	8.33 **	−10.67 **	48.68 **	−13.92 **	33.33 **		14.29 **
LSD 0.05	0.23	0.02	0.74	0.13	0.12	0.01		0.01
LSD 0.01	0.31	0.03	0.98	0.17	0.16	0.012		0.013

* and ** are the non-significant and significant effect at 0.05 and 0.01 probability.

**Table 6:** Eigen values, % variance, and cumulative % variance of 15 morphological traits for principal components.

Traits	PC1	PC2	PC3	PC4	PC5	PC6	PC7	PC8	PC9	PC10	PC11	PC12	PC13	PC14	PC15
Eigen value (Root)	7.55	2.26	1.65	1.13	0.77	0.57	0.41	0.28	0.16	0.10	0.04	0.03	0.02	0.01	0.01
Variance contribution (%)	50.35	15.07	11.00	7.50	5.16	3.82	2.73	1.90	1.06	0.66	0.27	0.21	0.15	0.06	0.05
Cumulative variance contribution	50.35	65.43	76.42	83.92	89.09	92.91	95.64	97.54	98.59	99.25	99.52	99.73	99.89	99.95	100.0
Days to heading (day)	0.19	0.69	−0.42	−0.14	0.79	0.63	0.38	0.93	0.93	0.95	0.49	0.97	−0.02	0.97	0.95
Plant height (cm)	−0.41	−0.58	−0.03	0.74	−0.03	−0.57	−0.32	0.10	0.10	0.17	0.80	0.11	0.24	0.13	0.13
Chlorophyll content (SPAD)	0.35	0.23	0.71	0.29	−0.05	−0.10	0.50	0.05	0.07	−0.10	0.00	0.01	0.77	0.03	0.00
Panicle length (cm)	0.76	0.02	0.01	−0.10	−0.30	−0.28	−0.53	0.17	0.18	−0.02	0.04	0.09	−0.07	0.04	−0.02
Panicle weight (g)	0.02	0.23	−0.03	0.47	−0.11	−0.25	0.31	0.12	0.16	−0.08	−0.18	0.06	−0.49	−0.02	−0.13
Flag leaf angle (°)	0.25	−0.04	−0.37	−0.13	0.31	−0.24	0.29	−0.06	−0.03	−0.14	0.22	−0.09	0.08	−0.05	−0.16
Flag leaf area (cm^2^)	0.02	−0.05	0.41	−0.15	0.30	−0.11	−0.02	−0.02	−0.04	0.02	0.15	−0.04	−0.29	0.04	−0.03
Panicle exsertion (%)	0.11	0.18	−0.05	0.26	0.24	0.08	−0.20	−0.15	−0.17	0.08	−0.05	−0.05	0.03	0.02	−0.01
Fertility percentage (%)	−0.05	0.16	−0.01	−0.09	−0.11	−0.18	0.03	−0.19	−0.10	0.11	0.04	0.10	0.00	0.07	0.05
1000-grain weight (g)	0.11	−0.11	0.00	0.05	−0.09	0.13	0.09	−0.12	−0.05	0.03	0.07	−0.02	−0.07	0.10	0.03
Number of panicles/plant	0.00	−0.05	−0.01	0.00	0.03	−0.06	0.00	−0.03	0.02	−0.05	−0.10	−0.05	0.01	0.08	0.11
Grain type	0.02	0.02	0.00	0.01	−0.02	0.00	0.02	0.05	−0.07	−0.01	0.03	−0.03	−0.02	−0.09	0.12
Grain yield t/ha	−0.02	0.07	0.00	−0.01	−0.03	0.02	−0.02	0.00	0.03	−0.06	0.06	−0.08	−0.01	0.04	0.00
Apparent heterosis	0.00	0.00	0.00	0.00	−0.01	−0.01	0.01	0.02	0.00	0.07	−0.01	−0.06	0.00	0.01	−0.01
Phenotypic acceptability	0.00	0.00	0.00	0.00	0.01	0.00	0.00	−0.05	0.06	0.01	0.00	−0.01	0.00	−0.04	0.02

**Table 7:** Origin, pedigree, aalience, and features of nine parental lines of hybrid rice.

No.	Entries	Pedigree	Origin	Salience and Features
1	Quan-9311A	222B/R9311//Zhong 9b	China	*Indica* type, medium maturing, semi dwarf, long grain, good grain quality, and CMS line (wide abortive)
2	Guang8A	Zengchengsimiao-8 Xuan/1325B (Yuefeng B/26)	China	*Indica* type, medium maturing, semi dwarf, long grain, good grain quality, and CMS line (wide abortive)
3	Heng Feng A	Xiangxuan/113B//zhenshan 97A	China	*Indica* type, medium maturing, semi dwarf, long grain, good grain quality, and CMS line (wide abortive)
4	II-32A	Zhenshan97/IR665	China	*Indica* type, CMS line, medium maturing, semi dwarf, long grain, good grain quality, and CMS line (wide abortive)
5	Quanxiang 9A	Zhenshan97/I-32B//58025B	China	*Indica* type, medium maturing, semi dwarf, short grain, good grain quality, and CMS line (wide abortive)
6	Giza179	GZ6296/GZ1368	Egypt	*Indica/Japonica* type, early maturing, semi dwarf, short grain, good grain quality, good restorer for CMS lines, and high yielder
7	Giza181	IR 28/IR 22	Egypt	*Indica* type, long maturing, semi dwarf, long grain, good grain quality, good restorer for CMS lines, and high yielder
8	Giza182	Giza181/IR39422-161-1-3-1/Giza181	Egypt	*Indica* type, medium maturing, semi dwarf, long grain, good grain quality, good restorer for CMS lines, and high yielder
9	GZ5121-5-2	GZ1368-5-S-4/LA110//Milyang49	Egypt	*Indica/Japonica* type, medium maturing, semi dwarf, short grain, good grain quality, good restorer for CMS lines, and high yielder.

## Data Availability

The data presented in this study are available in article.

## Supplementary Materials

The following are available online at https://www.mdpi.com/article/10.3390/plants11030266/s1, Table S1. Mean performance for grain yield and its contributing traits for lines, testers and their crosses.
