# Supplementary material for: Assessment of Genetic Parameters and Gene Action Associated with Heterosis for Enhancing Yield Characters in Novel Hybrid Rice Parental Lines
Source: Plants (Basel). 2022 Jan 19;11(3):266. doi: 10.3390/plants11030266 (PMC8838428; doi:10.3390/plants11030266)
Supplement: Supplementary file 1 [file plants-11-00266-s001.zip › plants-1495478-supplementary.pdf]

Table S1. Mean performance for grain yield and its contributing traits for lines, testers and their crosses.

	Genotypes	Days to Heading	Plant Height (cm)	Chlorophyll Content (SPAD)	Flag Leaf Angle (o)	Flag Leaf Area (cm^2)	Panicle Exsertion (%)	Panicle Length (cm)	Number of Panicles $Plant^{-1}$	Panicle Weight (g)	1000-Grain Weight (g)	Fertility (%)	Grain Yield t ha^{-1}	Grain Type	Apparent Heteroses	Phenotypic Acceptability
1	Quan-9311A	75.24	85.27	40.03	19.87	84.37	100.00	30.50	9.00	1.52	22.1	92.00	2.71	8.03	6.03	7.04
2	Guang8A	72.23	76.24	40.03	23.15	36.26	87.99	26.00	7.33	0.97	20.1	85.00	2.81	7.52	4.02	6.03
3	Heng Feng A	76.24	82.26	43.03	41.37	35.04	86.98	26.00	7.67	1.17	20.1	87.50	2.91	8.53	4.02	6.03
4	II-32A	92.29	84.26	40.03	30.20	43.13	83.94	19.50	8.67	1.33	21.1	91.00	3.01	5.02	5.03	6.03
5	Quanxiang 9A	78.24	83.26	42.03	40.03	42.37	85.97	28.33	8.00	1.21	21.1	89.00	3.01	7.92	5.03	6.03
6	Giza179	65.11	99.00	35.53	24.80	70.37	100.00	25.17	10.00	2.11	26.1	79.82	7.40	5.13	7.04	8.04
7	Giza181	89.77	113.00	41.03	6.63	60.27	100.00	27.85	9.06	1.62	22.1	69.85	5.43	8.88	6.03	7.04
8	Giza182	74.23	96.67	40.03	13.23	53.23	100.00	24.67	10.00	1.99	25.1	76.83	6.91	8.38	6.03	8.04
9	GZ5121	76.24	108.00	40.03	21.50	77.10	100.00	26.67	9.57	1.69	24.1	74.83	5.92	3.95	6.03	7.04
10	Quan-9311A × Giza181	87.11	108.33	42.63	18.20	64.87	99.11	27.00	12.33	3.89	26.7	89.80	9.86	7.40	8.04	8.04
11	Quan-9311A × Giza182	85.13	118.00	40.60	21.50	84.13	99.40	28.83	11.75	3.57	26.1	87.81	9.40	7.69	8.04	8.04
12	Quan-9311A × Giza179	86.27	115.59	38.57	23.13	77.50	100.00	28.00	17.33	5.77	28.1	93.79	13.26	6.63	9.05	9.05
13	Quan-9311A × GZ5121	91.29	122.45	42.03	10.07	29.40	100.00	29.86	7.00	0.83	17.1	10.00	0.32	4.61	3.02	5.03
14	Guang8A×Giza 179	76.24	116.33	39.03	29.77	69.50	99.11	28.33	10.00	1.87	24.5	74.83	6.02	8.03	6.03	7.04
15	Guang8A×Giza 181	92.83	132.67	38.03	13.23	91.20	98.10	31.33	13.75	4.39	27.5	91.80	12.04	8.23	9.05	9.05
16	Guang8A×Giza 182	80.54	114.67	41.62	25.16	55.48	100.00	28.00	10.67	2.87	26.1	84.81	9.03	8.53	8.04	8.04
17	Guang8A×GZ5 121	80.84	109.33	37.56	13.23	82.77	97.09	27.00	12.00	3.66	26.7	87.81	9.50	4.31	8.04	8.04
18	Heng Feng A×Giza179	78.86	104.63	35.53	16.53	59.43	100.00	25.50	12.67	4.00	27.1	89.80	10.03	4.71	8.04	8.04
19	Heng Feng A×Giza181	95.30	110.00	36.54	11.07	65.67	100.00	27.00	11.67	3.51	26.1	87.81	9.26	7.82	8.04	8.04
20	Heng Feng A×Giza182	79.19	111.12	40.03	18.20	70.97	99.40	27.70	13.33	4.33	27.1	89.80	10.53	6.42	9.05	9.05
21	Heng Feng A×GZ5121	81.17	102.67	34.02	21.57	71.87	99.11	25.33	11.00	3.27	26.1	86.81	9.11	6.02	8.04	8.04
22	II-32A×Giza179	83.15	123.00	38.57	21.53	70.07	99.40	30.20	14.09	4.63	27.7	91.80	12.04	5.22	9.05	9.05
23	II-32A×Giza181	89.09	114.33	39.59	35.23	71.45	100.00	28.00	15.19	4.87	28.1	92.80	13.04	8.03	9.05	9.05
24	II-32A×Giza182	86.58	119.67	40.60	31.47	75.53	96.08	29.17	10.67	3.17	26.1	84.81	9.03	8.23	8.04	8.04
25	II-32A×GZ5121	83.56	107.00	37.56	18.20	56.17	100.00	26.33	10.00	2.25	26.1	79.82	8.53	5.02	7.04	8.04

26	Quanxiang 9A ×Giza179	79.85	109.33	42.03	20.21	68.13	99.11	27.00	10.00	2.04	26.1	79.82	7.02	6.82	7.04	8.04
27	Quanxiang 9A ×Giza181	90.08	95.67	41.03	13.23	45.75	98.10	21.47	9.00	1.43	22.1	69.89	3.01	8.03	5.03	7.04
28	Quanxiang 9A ×Giza182	81.17	110.05	41.03	38.00	60.67	99.40	27.18	12.83	4.13	27.1	89.80	10.03	8.13	9.05	9.05
29	Quanxiang 9A ×GZ5121	82.16	105.00	42.63	13.23	70.17	100.00	26.00	10.67	2.53	26.1	79.82	8.73	6.82	8.04	8.04
	LSD 0.05	1.108	2.225	0.699	0.557	2.033	0.157	0.424	0.536	0.231	0.03	0.735	0.126	0.12	0.007	0.005
	LSD 0.01	1.47	2.96	0.93	0.74	2.71	0.21	0.56	0.71	0.31	0.03	0.98	0.17	0.16	0.012	0.013
